# ATP- and Polyphosphate-Dependent Glucokinases from Aerobic Methanotrophs

**DOI:** 10.3390/microorganisms7020052

**Published:** 2019-02-14

**Authors:** Alexander S. Reshetnikov, Natalia P. Solntseva, Olga N. Rozova, Ildar I. Mustakhimov, Yuri A. Trotsenko, Valentina N. Khmelenina

**Affiliations:** 1 Laboratory of Methylotrophy, G.K. Skryabin Institute of Biochemistry and Physiology of Microorganisms, Russian Academy of Sciences, Federal Research Center “Pushchino Scientific Center for Biological Research of the Russian Academy of Sciences”, Pushchino 142290, Russia; reshetnikovas@rambler.ru (A.S.R.); olgan.rozova@gmail.com (O.N.R.); mii80@rambler.ru (I.I.M.); trotsenko@ibpm.pushchino.ru (Y.A.T.); 2 Department of Microbiology and Biotechnology, Pushchino State Institute of Natural Sciences, Prospect Nauki 3, Pushchino 142290, Russia; natalia.solntseva.nn@gmail.com

**Keywords:** ATP-glucokinase, polyphosphate-glucokinase, methanotrophs, *Methylomonas* sp. 12

## Abstract

The genes encoding adenosine triphosphate (ATP)- and polyphosphate (polyP)-dependent glucokinases (Glk) were identified in the aerobic obligate methanotroph *Methylomonas* sp. 12. The recombinant proteins were obtained by the heterologous expression of the *glk* genes in *Esherichia coli*. ATP-Glk behaved as a multimeric protein consisting of di-, tri-, tetra-, penta- and hexamers with a subunit molecular mass of 35.5 kDa. ATP-Glk phosphorylated glucose and glucosamine using ATP (100% activity), uridine triphosphate (UTP) (85%) or guanosine triphosphate (GTP) (71%) as a phosphoryl donor and exhibited the highest activity in the presence of 5 mM Mg^2+^ at pH 7.5 and 65 °C but was fully inactivated after a short-term incubation at this temperature. According to a gel filtration in the presence of polyP, the polyP-dependent Glk was a dimeric protein (2 × 28 kDa). PolyP-Glk phosphorylated glucose, mannose, 2-deoxy-D-glucose, glucosamine and *N*-acetylglucosamine using polyP as the phosphoryl donor but not using nucleoside triphosphates. The *K*_m_ values of ATP-Glk for glucose and ATP were about 78 μM, and the *K*_m_ values of polyP-Glk for glucose and polyP_(n=45)_ were 450 and 21 μM, respectively. The genomic analysis of methanotrophs showed that ATP-dependent glucokinase is present in all sequenced methanotrophs, with the exception of the genera *Methylosinus* and *Methylocystis*, whereas polyP-Glks were found in all species of the genus *Methylomonas* and in *Methylomarinum vadi* only. This work presents the first characterization of polyphosphate specific glucokinase in a methanotrophic bacterium.

## 1. Introduction

Glucokinase (Glk, E.C. 2.7.1.63) catalyzes the phosphorylation of glucose into glucose 6-phosphate using nucleoside triphosphates and/or inorganic polyphosphate (polyP) as a phosphoryl donor. In prokaryotic and eukaryotic cells, Glk initiates the involvement of glucose in the central metabolic pathways. However, despite a wide distribution, the role of glucokinase in microorganisms is not quite clear. In *Escherichia coli* transporting glucose into cells via the phosphoenolpyruvate-dependent phosphotransferase system (PTS), Glk is not needed to support the growth on sugar as the deletion of the *glk* gene was not affected by the growth of the mutant strain [1,2]. A mutation analysis revealed that PTS is of major importance in permitting the rapid growth of *E. coli* on glucose It has been proposed that glucokinase in *E. coli* is necessary for the utilization of disaccharides consisting of glucose, such as lactose, maltose and trehalose, taken up by alternative mechanisms [1].

Aerobic bacteria utilizing methane as a carbon and energy source (methanotrophs) inhabit various natural systems where they mitigate this greenhouse gas emission [3]. Methanotrophs represent about 30 genera belonging to the phyla *Proteobacteria* and *Verrucomicrobia,* as well as to the candidate division NC10 [4,5,6]. Although all known methanotrophs are unable to grow on any sugars, most of them possess the Glk encoding genes [7]. ATP-dependent glucokinase (ATP-Glk) from the halotolerant methanotroph *Methylomicrobium alcaliphilum* 20Z has been biochemically characterized, and an essential role of the enzyme in the reutilization of free glucose derived from intracellular sucrose and glycogen has been revealed [7]. A preliminary genomic analysis showed that methanotrophs of the genus *Methylomonas* possess at least two glucokinase-like genes. However, the functioning of the glucokinase isozymes in methanotrophs has not been studied.

In the 1970s, the red pigmented obligate methanotroph was isolated from freshwater pool, and it has been previously described as “*Methylomonas methanica*” sp. 12 [8]. This bacterium has been intensively investigated as a potential producer of single cell proteins [9]. It was found that this methanotroph synthesizes inorganic polyphosphates, accumulating these compounds up to 6 mg/g of dry cells [10]. Here, we checked the phylogenetic position of this bacterium. In addition, two glucokinase encoding genes were identified and cloned, and recombinant enzymes were characterized. The distribution of polyphosphate dependent glucokinase in methanotrophs was analyzed.

## 2. Materials and Methods

### 2.1. Bacteria and Growth Conditions

The red pigmented obligate methanotroph “*Methylomonas methanica*” sp. 12 used in this study was previously isolated from a freshwater pool [8]. Now we sequenced the 16S rRNA encoding gene amplified by PCR with primers 27f/1492r [11] (Weisburg et al., 1991), as well as another taxonomically valuable gene *pmoA* coding for a large subunit of the particulate methane monooxygenase using primers A189f/mb661r [12,13]. (Holmes et al., 1995; Costello & Lidstrom, 1999) The 16S rDNA and *pmoA* sequences have been deposited in the GenBank under the accession numbers MK158248 and MK165450, respectively. The 16S rDNA sequencing analysis showed 98% similarity with *Methylomonas koyamae* Fw12E-YT (AB538964) and only 94.4% similarity with *Methylomonas methanica* S_1_^T^ (AF304196) (Figure S1). A phylogenetic analysis showed that the *pmoA* sequence from strain 12 branched within the *Methylomonas* group of *pmo*A sequences (Figure S2). This bacterium was designated as *Methylomonas* sp. 12. *Methylomonas* sp. 12 was grown at 30 °C under a methane and air mixture (1:1) in a mineral medium P at pH 6.8 [8]. *Escherichia coli* Rosetta (DE3), obtained from Stratagene (La Jolla, CA, USA), was grown at 30 °C in a liquid or solid (1.5% agar) LB medium [14] supplemented with 25 μg kanamycin when necessary.

### 2.2. Identification of Genes Coding for PolyP- and ATP-Dependent Glucokinases

In the published genomes of *Methylomonas* species, two glucokinase-like genes coding for putative ATP-Glk and polyP-Glk have been found. Based on these sequences, two pairs of degenerate primers (GluK(F)A12/GluK(R)A12 and PolyGK-R/PolyGK-F2) were designed to amplify the internal fragments of these genes using the DNA of *Methylomonas* sp. 12 as a template (Table S1). The PCR fragments obtained were sequenced and the missing 5′- and 3′-regions of each gene were identified by an inverse PCR. The genomic DNAs were broken up by endonucleases *EcoR*I and *Hind*III, and the DNA fragments were ligated to themselves by a T4 DNA ligase overnight at 16 °C. Following this, the DNA was precipitated from the ligase mixture by the addition of a 2.5 volume of ethanol and 0.1 volume of 3 M sodium acetate, before being centrifuged during 30 min at 14,000× *g*. The sediments were washed sequentially by 80% and 96% ethanol and dissolved in a Tris-EDTA buffer (10 mM tris(hydroxymethyl)aminomethane-HCl, 1 mM ethylenediaminetetraacetate, pH 8.0). The circular DNA molecules were used as a matrix in an inverse PCR. Based on the DNA fragments sequences, two pairs of the complementary primers GluKA12-F1/GluKA12-R1 and F2polGK/RpolGK were constructed for inverse PCRs to identify the 5′- and 3′-regions of ATP- and polyP-dependent glucokinase encoding genes, respectively (Table S1). The obtained PCR products were sequenced and analyzed. The complete sequences of the *glk* and *pglk* genes coding for ATP-Glk and polyP-Glk were deposited into the Database NCBI (#MH925106 and #MH925105, respectively).

### 2.3. Cloning of Glucokinases Encoding Genes

The gene *glk* encoding putative ATP-Glk was amplified from the genomic DNA by the primers ATP-Glk-F and ATP-Glk-R (Table S1). The PCR product was treated by the endonucleases *Nde*I and *Sal*I and ligated in the expression vector pET30(a)+ (Novagene), treated by the same restrictases. The entire polyP-Glk encoding gene (*pglk*) was amplified by using the primers Pol-Glk-F and Pol-Glk-R (Table S1), and the PCR product was treated by the endonucleases N*de*I and *Xho*I. The resulting plasmids pET-polyP-glk and pET-ATP-glk were transferred to *E. coli* Rosetta (DE3), and the expression of the proteins was induced by 0.5 mM isopropyl β-D-1-thiogalactopyranoside (Sigma-Aldrich, St. Louis, MO, USA) added in a logarithmic phase of growth (А_600_ = 0.6–0.8). The cells were incubated during 18 h at 20 °C and centrifuged for 20 min at 6000 g.

### 2.4. Purification of the Recombinant Proteins

0.5 g of transformed *E. coli* cells were re-suspended in 8 mL of 50 mM Tris-HCl, pH 8.0, containing 500 mM NaCl and 5 mM imidazol, and disrupted in a disintegrator Sonicator S-4000 (USA) for 10 min with 30 s cooling in ice after each 15 s of sonication. The homogenates were centrifuged for 30 min at 11,000× *g* and 4 °C, and the supernatant was transferred into a column with Ni^2+^-NTA-agarose (Quiagen, Germany), equilibrated with the same buffer. The column was washed by three volumes of buffer B (50 mM Tris-HCl, pH 8.0, containing 500 mM NaCl and 60 mM imidasol). The protein was eluted by a buffer B containing 200 mM imidazol. The proteins in the fractions were identified by sodium dodecyl sulfate–polyacrylamide gel electrophoresis (SDS-PAGE) [15]. The fractions containing proteins were dialyzed against a 100 mM Tris-HCl buffer, pH 7.5, with 200 mM NaCl.

### 2.5. Determination of Molecular Masses of Glucokinases

The native masses of the recombinant N-terminal His-tagged glucokinases were determined by electrophoresis in a gradient of polyacrylamide gel (4–30%) in a buffer solution (pH 8.4) containing 190 mM glycine and 90 mM Tris-HCl [16]. After a preliminary electrophoresis during 15 min at 70 V, the samples were re-dissolved in the same buffer supplemented by 10% glycerol and 0.5 μL 1% bromphenol blue and electrophoresed again at 150 V during 15 h. Thyroglobulin (669 kDa), ferritin (440 kDa), amylase (232 kDa), alcohol dehydrogenase (140 kDa), and bovine serum albumin (BSA) (67 kDa) (Sigma, USA) were used as protein markers.

The molecular masses of the glucokinases were also determined by a size exclusion chromatography using AKTAstart on a Tricorn Superdex 200 10/300 GL column (GE Healthcare) calibrated with the gel filtration standards purchased from Bio-Rad (Thyroglobulin 670 kDa; γ-globulin, 158 kDa; ovalbumin, 44 kDa; myoglobin, 17 kDa; Vitamin B12, 1,3 kDa). As a running buffer 100 mM Tris-HCl (pH 8.0), 100 mM NaCl, and 5 mM MgCl_2_ were used at a flow rate of 0.8 mL/min. 10 mM polyP was added, if necessary, to the running buffer. Pure ATP-Glk-His_6_ or polyP-Glk-His_6_ (10 mg) was loaded onto the column. Fractions of 0.5 mL were collected. Aliquots from these fractions were tested for glucokinase activities and analysed by SDS-PAGE.

### 2.6. Essay of Enzyme Activities

To determine the activity of the ATP-Glk, the reduction of nicotinamide adenine dinucleotide phosphate (NADP^+^) was registered in 1 mL of the reaction mixture containing 50 mM K-phosphate buffer, pH 7.5, 5 mM ATP, 5 mM MgCl_2_, 0.5 mM NADP^+^, 5 mM glucose, and 5 U glucose 6-phosphate dehydrogenase (GPD) at 340 nm on a spectrophotometer Shimadzu UV-1700 (Japan). The activity of the polyP-Glk was measured at 28 °C in 1 mL of a reaction mixture containing 50 mM Tris-HCl buffer, pH 8.5, 5 mM glucose, 2.5 mM MgCl_2_, 0.5 mM NADP^+^, 5 U GPD, and 0.05 mM polyP (Type 45, Sigma-Aldrich, USA).

The specificity of ATP-Glk to sugars (D-glucose, D-mannose, D-galactose, 2-deoxy-D-glucose, glucosamine, N-acetylglucosamine, sucrose, maltose, trehalose, L-arabinose, D-xylose, ribose 5-phosphate, fructose 6-phosphate, glucose 6-phosphate or glucose 1-phosphate (Sigma-Aldrich, USA)) was determined in the reaction mixture containing a 50 mM buffer: 5 mM ATP, 25-50 mM MgCl_2_, 5 mM phosphoenolpyruvate, 0.25 mM NADH, 5 U pyruvate kinase, and 5 U lactate dehydrogenase. The reaction was started by adding sugar (5 mM). The sugar specificity of polyP-Glk was determined by testing a decrease of the polyP concentration [17]. The reaction mixture (0.1 mL) contained 50 mM of Tris-HCl buffer, pH 8.5, 0.1 mM polyP, 25 mM MgCl_2_, 15 mM sugar (D-glucose, D-mannose, D-galactose, 2-deoxy-D-glucose, glucosamine, N-acetylglucosamine, sucrose, maltose, trehalose, L-sorbose, D-xylose, lactose) and ~80 μg polyP-Glk. After 10 min of incubation, a 10 µL aliquot was placed into 1 mL of solution (6 mg of toluidine blue in 1 L of 40 mM acetic acid). The concentration of polyphosphate was calculated from the optical density ratio at 530/630 nm using the respective concentration of polyP as a standard. The absorption spectra were registered in a spectrophotometer Shimadzu UV-160 (Japan).

### 2.7. Biochemical Characterization of Glucokinases

To study the pH dependence of the enzyme activities, the following buffers were used (50 mM): MES-NaOH (pH 5.5–6.0), K-phosphate (pH 6.0–8.0), Tris-HCl (pH 7.6–8.9), CHES-NaOH (9.0–10.0); and glycine-NaOH (9.0–10.5). To check the temperature optima, the reactions were performed in the temperature range of 10 to 70 °C. To study the thermostability, the enzyme aliquots were heated for 5–180 min at 30–65 °C, and the residual activities were measured in the standard conditions. The effect of metals was tested using the aqueous solutions of ZnCl_2_, CaCl_2_, BaCl_2_, MnCl_2_, CoCl_2_, CdCl_2_, SrCl_2_, CuCl_2_, NiCl_2_, CsCl, RbCl or LiCl (at final concentrations of 5 or 1 mM), and of NaCl or KCl at different concentrations. The specificity to the phosphoryl donor was investigated in the standard reaction mixture where ATP was replaced by cytosine triphosphate (CTP), GTP, UDP or adenosine diphosphate (ADP) (5 mM), by inorganic pyrophosphate (PPi) or tripolyphosphate (PPPi) (2–3 mM) as well as by polyP_45_ (0.1 mM). *K_m_* and *V_max_* were calculated from the enzyme activity measured at various concentrations of one substrate and the saturated concentration of another. The ranges of the glucose concentration were 0.161–20 mM; for ATP, they were 0.161–10 mM, and they were 0.4–6.25 µM for polyP.

### 2.8. Analysis of Nucleotide and Amino Acid Sequences

The comparative analyses of protein sequences were performed by using PSI-BLAST (http://www.ncbi.nlm.nih.gov). The analysis of nucleotide sequences, determination of restriction sites and open reading frames were performed by using program VectorNTI v.9.0. The alignment of amino acid sequences was performed via ClustalX (v1.62b) [18]. The phylogenetic analysis was conducted by using MEGA6 (model Neighbor-Joining) [19].

## 3. Results

### 3.1. Identification of Glucokinase Encoding Genes in Methylomonas sp. 12 and Purification of Glucokinases

The crude sonic extracts of *Methylomonas* sp. 12 catalyzed a glucose phosphorylation into glucose 6-phosphate in the presence of either ATP or polyphosphate with an activity of 0.054 and 0.0058 U/mg of protein, respectively. By using inverse PCR, two genes encoding glucokinase isozymes were identified in the DNA of *Methylomonas* sp. 12. One gene of 990 b.p. encoded putative Glk sharing a 67% identity to the recently characterized ATP-Glk from the halotolerant methanotroph *Mm. alcaliphilum* 20Z [7], while another gene of 795 b.p. encoded an enzyme mostly similar (40% identity) to polyP-Glk from *Propionibacterium freudenreichii* [20].

**Figure 1:**
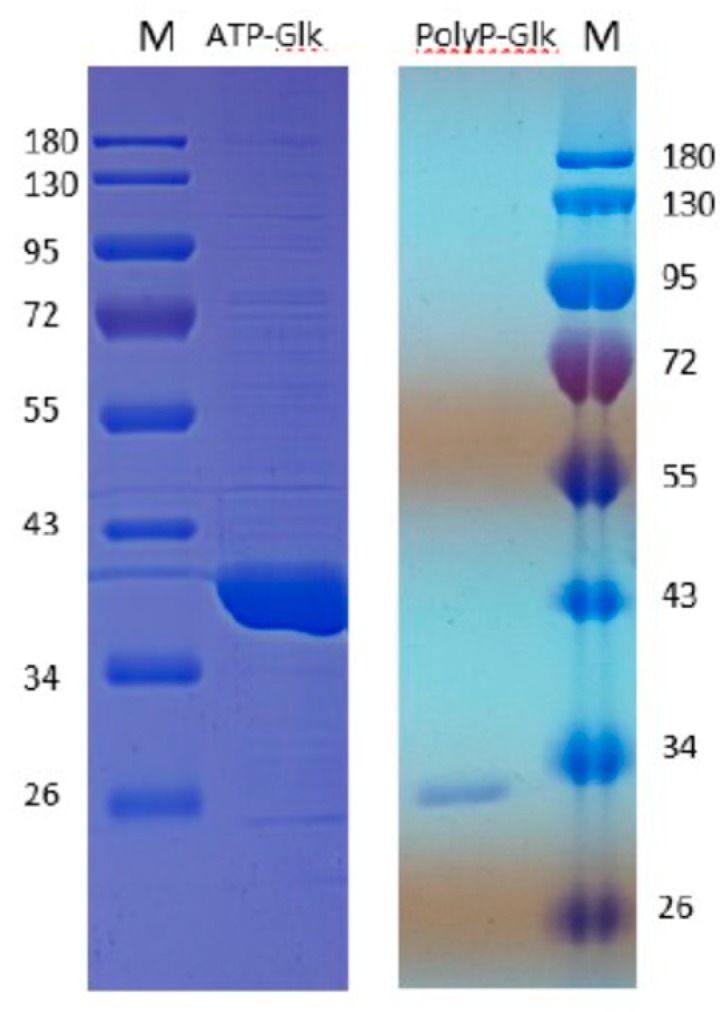
12% sodium dodecyl sulfate–polyacrylamide gel electrophoresis of 100 μg recombinant ATP-dependent glucokinase and 20 μg polyP-dependent glucokinase from *Methylomonas* sp. 12. M-markers of molecular masses, kDa.

The SDS-PAGE of the proteins obtained by the heterologous expression of the *glk* and *pglk* genes in *Esherichia coli* showed one protein band of about 35 kDa for ATP-Glk and 30 kDa for polyP-Glk (Figure 1). These values were in good agreement with the theoretically calculated subunit sizes of 35.5 kDa and 28.1 kDa.

The gradient electrophoresis of ATP-Glk-His_6_ in non-denaturation conditions revealed several protein bands of ~71 kDa (dimer), 106.5 kDa (trimer), 142 kDa (tetramer), 177.5 kDa (pentamer) and 213 kDa (hexamer). The structure of the enzyme was differed from that of *Mm. alcaliphilum* ATP-Glk which was a homodimeric enzyme [7]. According to the gel-filtration, the *Mr* of polyP-Glk was ~28 kDa, which doubled to ~60 kDa when polyP was added to the enzyme. The dimerization of the enzyme in the presence of polyphosphate has been previously reported for polyP/ATP-dependent Glk from *Corynebacterium glutamicum* [21]. Most the polyP-Glks investigated so far were either monomeric with a molecular weight of 30-50 kDa, or homodimeric [22,23].

### 3.2. Biochemical Properties of ATP-Glk

**Figure 2:**
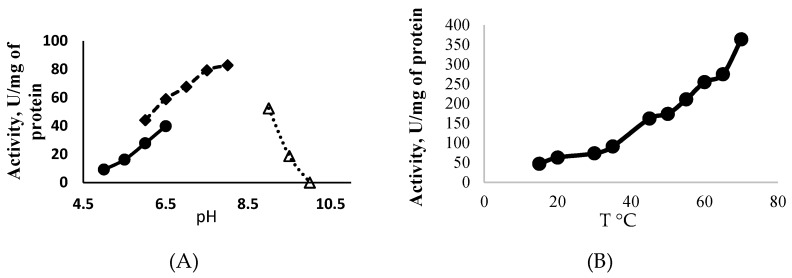
Effect of the pH (**A**) and the temperature (**B**) on the activity of ATP-dependent glucokinase from *Methylomonas* sp. 12.

ATP-Glk from *Methylomonas* sp. 12 catalyzed the phosphorylation of glucose in the presence of ATP (100% of activity), UTP (85%) and GTP (71%), but CTP, ADP, PPi or polyP did not serve as donors of the phosphoryl groups. ATP-Glk was active in a wide pH range (from pHs 5.0 to 9.5) with a maximum activity at pH 7.5, but its activity was sharply lost at pH > 9.0 (Figure 2A). It displayed the highest activity at 70 °C (Figure 2B) but fully inactivated after a 5 min incubation at 65 °C.

ATP-Glk phosphorylated glucose and glucosamine but other sugars (see Material and Methods section) did not serve as acceptors of the phosphoryl groups. The enzyme activity was dependent on divalent metals: Mg^2+^ (100% activity), Mn^2+^ (60%), Sr^2+^ (30%), Co^2+^ (18%), Ni^2+^ (13%), Cd^2+^ (11%), Cu^2+^ (3%). The highest activity was observed in the presence of 5 mM of MgCl_2_. It also displayed activity if Mg^2+^ was replaced by monovalent metal ions at a concentration of 5 mM: Li^+^ (72% of activity with Mg^2+^), Rb^+^ (73%), Cs^+^ (52%), or Na_2_MoO_4_ (66%). When NaCl (0.5 M) was added into the reaction mixture, the enzyme lost 50% of its activity. ADP inhibited the enzyme activity, while another reaction product, glucose 6-phosphate, had no appreciable effect on its activity (Table 1). A similar effect of ADP was reported for ATP-Glk from *Mm. alcaliphilum* 20Z [7].

The substrate saturation curves of ATP-Glk obeyed the Michaelis-Menten equation. At 30 °C and pH 7.5, the *K*_m_ values for the glucose and ATP were: 0.08 ± 0.007 and 0.078 ± 0.0056 mM, respectively (Table 2). The *V*_max_ values with glucose, glucosamine and ATP were 92.5 ± 1.8, 94.4 ± 1.9 and 106.4 ± 2.4 U/mg of protein, respectively. Glk showed a high catalytic efficiency, since the ratio of *k*_cat_/*K*_m_ was 82.09 for glucose and 96.85 for ATP.

### 3.3. Biochemical Properties of PolyP-Glk

**Figure 3:**
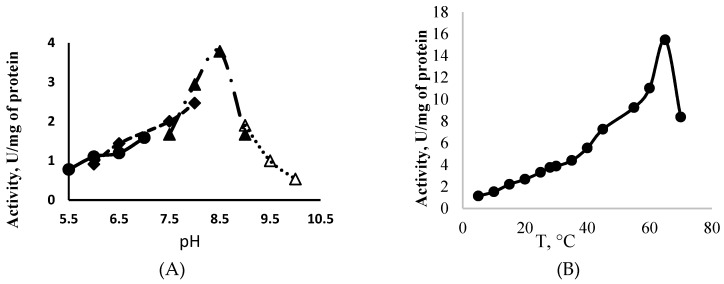
Effect of the temperature (**A**) and the pH (**B**) on the activity of polyphosphate-dependent glucokinase from *Methylomonas* sp. 12.

PolyP-Glk from *Methylomonas* sp. 12 was active at a pH range of 5.5–10.5, displaying a maximum activity at pH 8.5 and temperature 65 °C (Figure 3A,B). The alkaline pH optima have been found for polyP-Glk from *C. glutamicum* and *Thermobifida fusca* [21,24]. PolyP-Glk from *Methylomonas* sp. 12 was completely inactivated after heating for 5 min at 60–65 °C or after 30 min at 55 °C. After two hours of incubation at 35 °C and 40 °C, the residual activities were 36% and 5% of the initial activities. After a 2 h exposure at 28 °C, the enzyme retained more than 70% of its activity. High temperature optima were found for polyP-Glk from *Thermobifida fusca* (55 °C) and polyphosphate/ATP-glucomannokinase from *Arthrobacter* sp. KM (50 °C) [25].

PolyP-Glk from *Methylomonas* sp. 12 did not use ATP, GTP, CTP, UTP, ADP, PPi or PPPi as phosphoryl donors. So far, only three glucokinases have been described as having a strong specificity to polyphosphates: the enzyme from *Anabaena* sp. PCC 7120 (All1371), from *Microlunatus phosphovorus* [26,27], and that from mammalian hepatocytes [28]. Most other characterized polyP-Glks also used ATP, and/or other nucleoside triphosphates or ADP.

Besides glucose, polyP-Glk from *Methylomonas* sp. 12 phosphorylated mannose, 2-deoxy-D-glucose, glucosamine and N-acetylglucosamine, but did not use fructose, galactose, arabinose, L-sorbose, xylose, trehalose, ribose, sucrose lactose, glucose 1-phosphate, glucose 6-phosphate, fructose 1-phosphate or fructose 6-phosphate as an acceptor of the phosphoryl group. Its activity was dependent on divalent metal ions: Mg^2+^ (100% activity), Mn^2+^ (80%), Co^2+^ (79%), Cd^2+^ (44%), Ca^2+^ (13%) or Ni^2+^ (10%). It had a negligible activity if Mg^2+^ was replaced by 1 mM of monovalent metals: Li^+^ or Rb^+^ (3.5%), or Cs^+^ (2%). The cations Co^2+^, Ba^2+^, Ca^2+^, Zn^2+^ and Cu^2+^ did not significantly influence the enzyme activity in the presence of Mg^2+^. The highest activity was observed in the presence of 2.5 mM of MgCl_2_ and 100 mM of NaCl. At 28 °C and pH 8.5, the *K*_m_ values were: 0.45 ± 0.12 mM for glucose, and 0.021 ± 0.0039 mM for polyP (Table 2). The *V*_max_ were 7.04 ± 0.39 U/mg of protein for glucose, and 7.28 ± 0.45 U/mg of protein for polyP.

The activity of polyP-Glk was increased in the presence of 10 mM of ATP (Figure S3). An activating effect of ATP on polyP-dependent glucokinases has not been described previously. In contrast, competitive inhibition by high concentrations of ATP has been revealed for the *Mycobacterium tuberculosis* polyP-Glk that formed an enzyme-ATP-ATP complex [29]. Glucose 6-phosphate had no apparent effect on the polyP-Glk activity.

### 3.4. Distribution of Glucokinases in Methanotrophs

**Figure 4:**
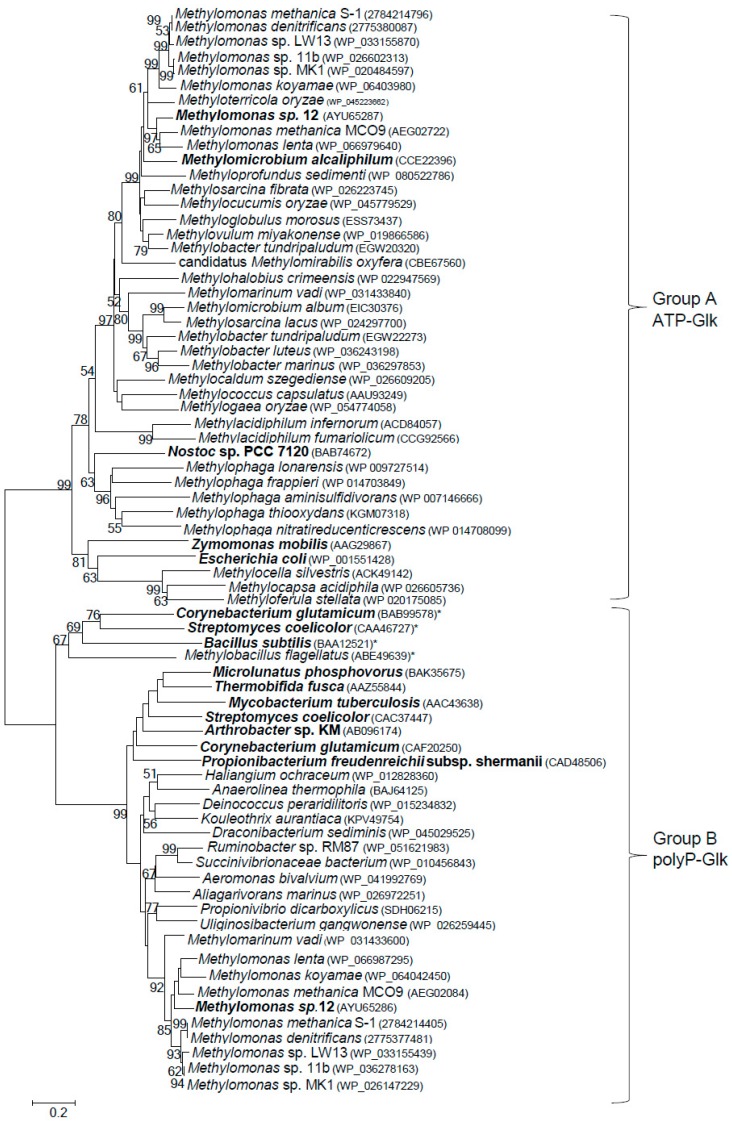
Phylogenetic tree constructed from the amino acid sequences of various putative and characterized bacterial ATP- and polyP-dependent glucokinases. The characterized enzymes are in bold and the amino acid accession numbers are in brackets. ATP-dependent glucokinases in the Group B are marked by asterisks. The scale bar corresponds to the number of substitutions per site.

ATP-Glk and polyP-Glk from *Methylomonas* sp. 12 shared a negligible similarity (only a 15% identity of amino acid sequences, IAA). The genes for putative ATP-Glk were found in genomes of all methanotrophs known to date, with exception of alphaproteobacterial methanotrophs of the *Methylosinus* and *Methylocystis* genera from the *Methylocystaceae* family (Figure 4). The methanotrophic *glk* genes are well conserved (48–70% of IAA). ATP-Glks of *Methylomonas* sp. 12 were mostly similar to those from *Methylomonas methanica* S_1_ and *Methylomonas lenta* (74–79% IAA) but had only 32–33% IAA with glucokinases from *E. coli*, *Zymomonas mobilis* (group A of glucokinases) and from *Bacillus subtilis* (group B of glucokinases) [30,31].

The *pglk*-like genes were found in all methanotrophs of the *Methylomonas* genus sequenced to date and also in *Methylomarinum vadi*, another gammaproteobacterial methanotroph. In the NCBI database, these genes were usually annotated as the ROK (Repressor, ORF, Kinase) proteins. However, the methanotrophic polyP-Glks do not possess the conserved sequence CXCGXXGCXE containing three cysteine residues occurring in the ROK family proteins which were found for the first time in the *Bacillus subtilis* ATP-Glk (GenBank NP_390365) [22].

## 4. Discussion

In this paper we have demonstrated for the first time that the obligate methanotroph *Methylomonas* sp. 12 expresses two functional (ATP- and polyP-dependent) glucokinases. ATP-dependent glucokinase is present in all sequenced methanotrophs, with the exception of the genera *Methylosinus* and *Methylocystis*, whereas polyP-Glks have been found only in methanotrophs of the genus *Methylomonas*, as well as in *Methylomarinum vadi*. There is an appreciable homology between methanotrophic ATP-Glks clustering together on the phylogenetic tree. The polyP-Glks of methanotrophs also comprise a coherent group of proteins, whereas ATP- and polyP-dependent glucokinases are highly divergent enzymes (Figure 4).

There is a rather high sequence identity (67%) between ATP-Glks from *Methylomonas* sp. 12 and that from another gammaproteobacterial methanotroph, *Mm. alcaliphilum* 20Z [7]. These enzymes also display similar biochemical properties, phosphorylating glucose and glucosamine and showing close temperature optima (60–65 °C), as well as instability at these temperatures. ADP inhibited the activities of both methanotrophic ATP-dependent glucokinases. The high affinity to glucose suggests that the enzyme from *Methylomonas* sp. 12, similar to the *Mm. alcaliphilum* ATP-Glk, can be involved in the scavenging of free glucose formed during the cleavage of intracellular glycogen [7]. Glycogen-like inclusions have been earlier demonstrated in the cells of *Methylomonas* sp. 12 [8]. Furthermore, it has been proven that exogenous glucose can enter the cells of *Methylomonas* sp. 12 and be an additional carbon source, since about 17% of cellular carbon originated from sugar during the culture growth under methane in the presence of 3 mM of radiolabeled glucose [32].

In contrast to ATP-Glk, polyP-Glk from *Methylomonas* sp. 12 demonstrated a strong specificity to polyP and a high *K*m value for glucose (450 μM). It also uses a wider range of acceptors of phosphoryl groups and, therefore, different physiological functions of the two enzymes in this bacterium can be suggested. In *Anabaena* sp. PCC 7120, the switching of the ATP-dependent glucokinase to the polyP-dependent enzyme in response to nitrogen starvation has been revealed. In mature heterocysts of the cyanobacterium, polyP-Glk can be involved in saving ATP for nitrogen fixation [27]. We hypothesize that two Glks in *Methylomonas* and *M. vadi* are also involved in overcoming environmental stresses. Importantly, *M. vadi* was isolated from a shallow submarine hydrothermal system, where it can be exposed to stressful conditions. In addition, *Methylomonas* and *Methylomarinum* are rather related genera in the context of the phylogeny of the PmoA-encoding genes (Figure S2) and of the similarity of other biochemical features distinguishing them from other gammaproteobacterial methanotrophs. For example, they have similar isoprenoid quinone compositions and possess phosphoenolpyruvate carboxylase, which is absent in other gammaproteobacterial methanotrophs [33,34]. The specificity of polyP-Glk towards glucose, mannose, 2-deoxy-D-glucose, glucosamine and *N*-acetylglucosamine (presumably formed during cell lysis in microbial communities or autolysis) may imply the participation of the enzyme in the survival of *Methylomonas* sp. 12 under methane limitation conditions.

Interestingly, among methanotrophs, only members of the family *Methylocystaceae* (the genera *Methylosinus* and *Methylocystis*) lack the genes for Glk. However, a Blast analysis revealed that the alphaproteobacterial methanotrophs of both *Methylocystaceae* and *Beyerinkiaceae* families possess homologues of the genes encoding the putative phosphoenolpyruvate phosphotransferase system, thereby implying that all methanotrophs are potentially able to use glucose as an additional carbon source. Altogether, our observations promote our understanding of the metabolic flexibility of obligate methanotrophs, including their high survival potential in nature, but again raise a long-standing question about the biochemical basis of their obligate dependence on C1-compounds.

## Figures and Tables

**Table 1:** Effect of different metabolites on the activities of ATP- and polyP-dependent glucokinases from *Methylomonas* sp. 12.

Effector, mM	ATP-Glk *	PolyP-Glk **
Control	100 *	100 **
Phosphoenolpyruvate, 5	99.8	111.3
Pyruvate, 5	114.9	103.3
Fructose-1-phosphate, 5	78.8	101.4
Fructose-6-phosphate, 5	115.8	103.0
Fructose-1,6- phosphate, 5	101.6	101.4
Glucose-1- phosphate, 5	101.7	107.2
Ribose-5-phosphate	nt	117.8
Adenosine triphosphate	100	155.0
Adenosine diphosphate, 5	48.9	93.0
Adenosine monophosphate, 5	110.3	84.1
KH_2_PO_4_, 3	86.1	nt
Glycerate, 1	109. 0	nt
Oxaloacetate, 1	108.8	105.3
α-Ketoglutarate, 1	108.8	99.7
Malate, 1	111.9	101.6
Citrate, 1	76.6	106.7
Isocitrate, 1	115.7	nt
Succinate, 1	114.9	nt
Polyphosphate, 0.075	117.3	100
Inorganic pyrophosphate, 2	82.3	104.0
Glyceraldehyde-3-phosphate	nt	111.6

* the reaction with ATP and glucose as substrates; ** the reaction with polyphosphate (n = 45) and glucose as substrates; nt not tested. The average values of three repeats of each reaction are presented.

**Table 2:** Kinetic parameters of ATP- and polyphosphate-dependent glucokinases from *Methylomonas* sp. 12.

Parameter	Substrate	Values	
		ATP-Glk	PolyP-Glk
*V_max_* (U/mg)	Glucose	92.5 ± 1.8	7.04 ± 0.39
	Glucosamine	94.4	
	ATP	106.4 ± 2.4	-
	PolyP_(n=45)_	-	7.28 ± 0.45
*K_m app_* (mM)	Glucose	0.08 ± 0.007	0.45 ± 0.12
	ATP	0.078 ± 0.0056	-
	PolyP _(n=45)_	-	0.021 ± 0.0039
*k_cat_* substrate (1/min)	Glucose	6.57	0.395
	ATP	7.55	-
	PolyP_45_	-	0.409
*k_cat_/K_m_* (1/min mM)	Glucose	82.09	0.87
	ATP	96.85	-
	PolyP _(n=45)_	-	19.476

## Supplementary Materials

The following are available online at http://www.mdpi.com/2076-2607/7/2/52/s1. Figure S1: The phylogenetic position of *Methylomonas* strain 12 among methanotrophs of the *Gammaproteobacteria* class and the genus *Methylomonas*. The methanotroph of the class *Alphaproteobacteria*, *Methylocystis parvus*, was used as an outgroup. Accession numbers are given in parentheses. Bar, 0.02 substitutions per nucleotide position; Figure S2: Phylogenetic analysis of the derived PmoA sequence from *Methylomonas* sp. 12. Bar, 0.02 substitutions per nucleotide position. The methanotroph of the class *Alphaproteobacteria*, *Methylocystis parvus*, was used as an outgroup; Figure S3: Effect of ATP on activity of polyP-Glk from Methylomonas sp. 12. PolyP Glk does not use ATP as a substrate in the absence of polyP.
