# Supplementary material for: ATP- and Polyphosphate-Dependent Glucokinases from Aerobic Methanotrophs
Source: Microorganisms. 2019 Feb 14;7(2):52. doi: 10.3390/microorganisms7020052 (PMC6406325; doi:10.3390/microorganisms7020052)
Supplement: Supplementary file 1 [file microorganisms-07-00052-s001.pdf]

Supplementary Material

ATP- and Polyphosphate-Dependent Glucokinases from Aerobic Methanotrophs

Table 1. Primers used in the work.

Primer	Sequences 5′-3′	Gene targeting
PolyGK-R	TT(R)AATTT(R)TCGAA(Y)TT(Y)TT	*pglk* (degenerate primer)
PolyGK-F2	TT(Y)AT(H)GGCAC(Y)(R)AT ATCGA	*pglk* (degenerate primer)
R-polGK CG	GCATCGGCGTCATTCAGGTT	*pglk* (for inverse PCR)
F2-polGK	ACGCTGGGGCAACCGCTTCAAC	*pglk* (for inverse PCR)
GluK(F)A12	ATGATTCTAGCCGGCGACAT	*glk* (degenerate primer)
GluK(R)A12	CAAAGTAATGCATGGCSCCAAKCAA	*glk* (degenerate primer)
GluKA12-F1	CGGCGACTTTATGCAGGCATT	*glk* (for inverse PCR)
GluK12-R1	CGATAGGATGATGCTGCTTGCCA	*glk* (for inverse PCR)
ATP-GlK-F	TCATATGATTCTTGCCGGCGACATAGGCGGT	*glk* (for cloning)
ATP-GlK-R	TGTCGACATCGGCTTGAAAATAATGCGCGGC	*glk* (for cloning)
Pol-Glk-F	TCATATGCGG ATTCTCGGCGTAGAT	*pglk* (for cloning)
Pol-Glk-R	TCTCGAGCGGGGCAAATGCCTCTT	*pglk* (for cloning)

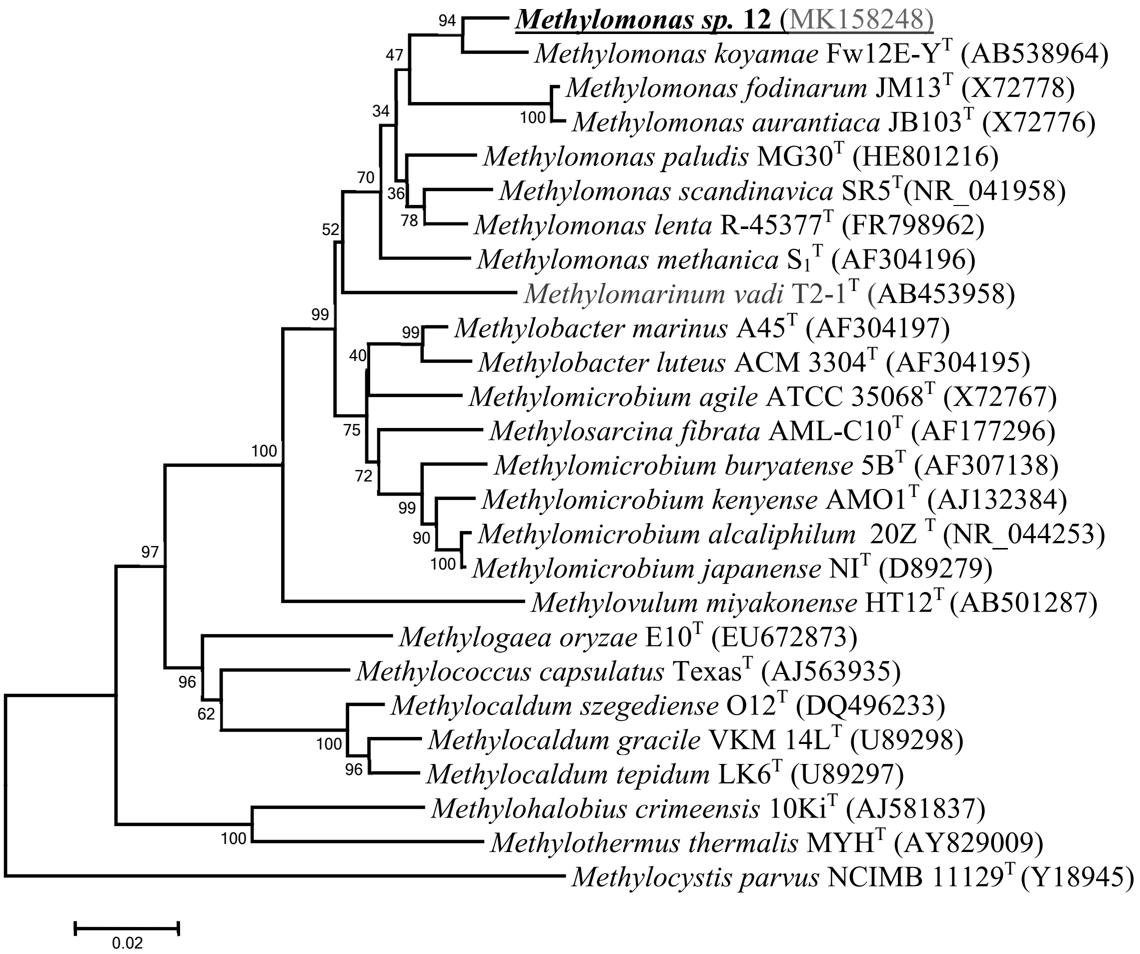

Figure S1. Phylogenetic position of *Methylomonas* strain 12 among methanotrophs of the *Gammaproteobacteria* class and the genus *Methylomonas*. The methanotroph of the class *Alphaproteobacteria*, *Methylocystis parvus*, was used as an outgroup. Accession numbers are given in parentheses. Bar, 0.02 substitutions per nucleotide position.

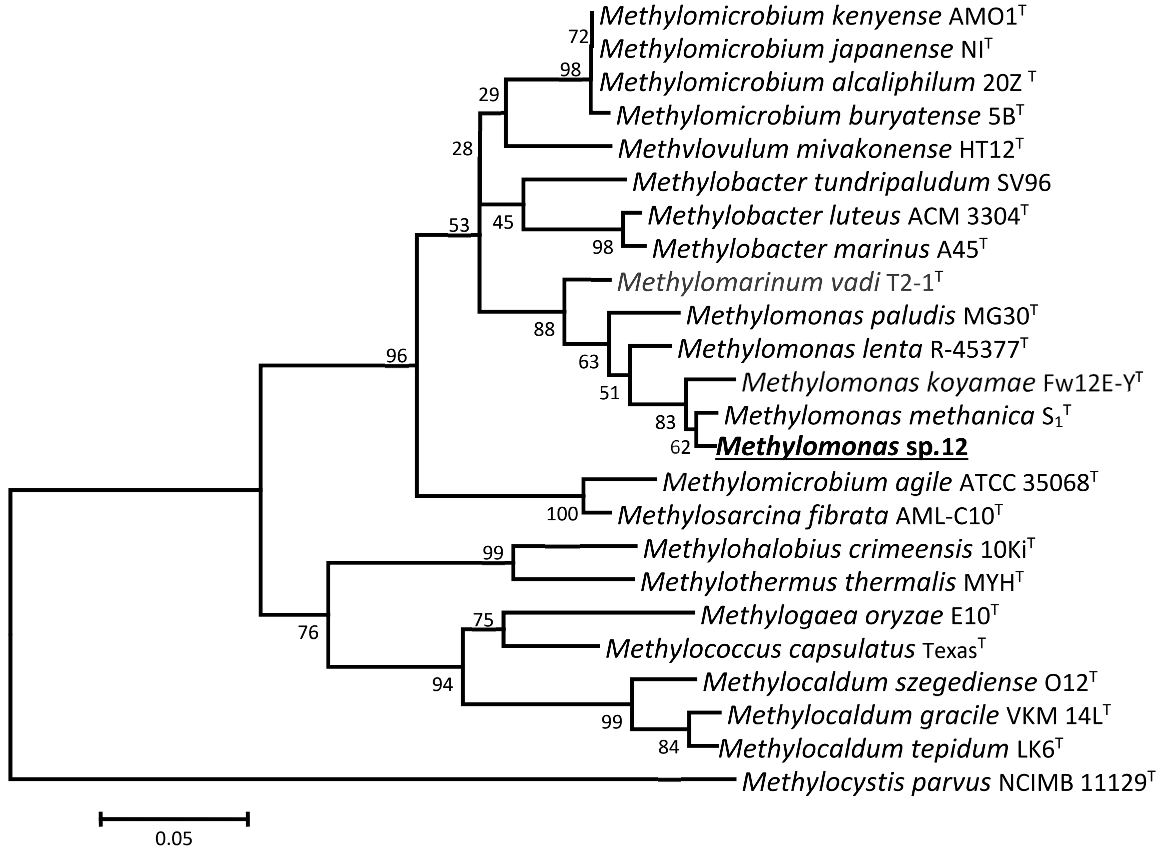

Figure S2. Phylogenetic analysis of the derived PmoA sequence from *Methylomonas* sp. 12. Bar, 0.02 substitutions per nucleotide position. The methanotroph of the class *Alphaproteobacteria*, *Methylocystis parvus*, was used as outgroup.

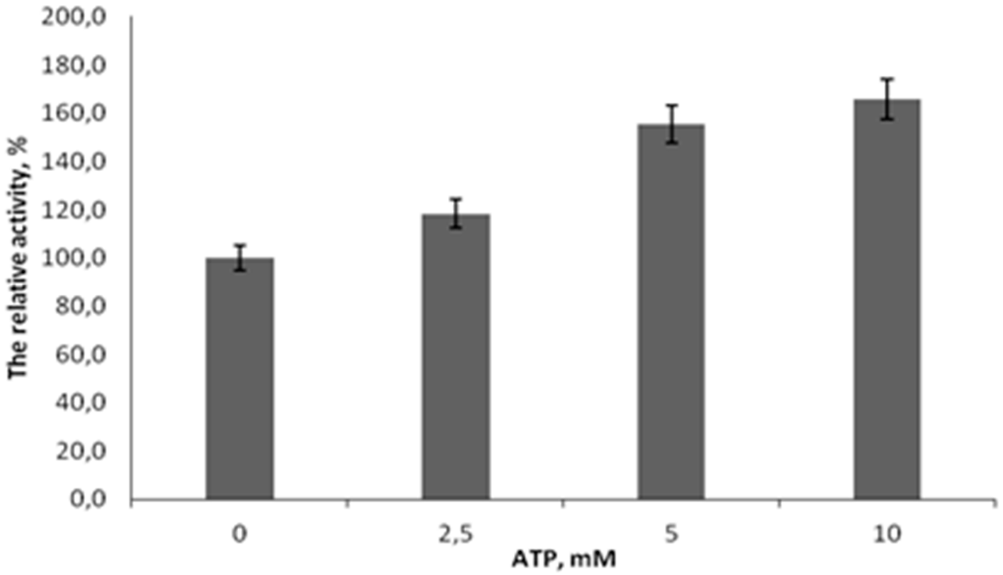

Figure S3. Effect of ATP on activity of polyphosphate-dependent glucokinase from *Methylomonas* sp. 12. The enzyme does not use ATP as a substrate in the absence of polyphosphate.
